# Efficacy, humoral, and cell-mediated immune response of inactivated fowl adenovirus 8b propagated in chicken embryo liver cells using bioreactor in broiler chickens

**DOI:** 10.14202/vetworld.2022.2681-2692

**Published:** 2022-11-26

**Authors:** Chidozie Clifford Ugwu, Mohd Hair-Bejo, Mat Isa Nurulfiza, Abdul Rahman Omar, Aini Ideris

**Affiliations:** 1 Department of Pathology and Microbiology, Faculty of Veterinary Medicine, Universiti Putra Malaysia, Serdang 43400, Selangor, Malaysia; 2 Department of Animal Science and Technology, Federal University of Technology, Owerri 460114, Imo State, Nigeria; 3 Laboratory of Vaccines and Biomolecules, Institute of Bioscience, Universiti Putra Malaysia, Serdang 43400, Selangor, Malaysia; 4 Department of Cell and Molecular Biology, Faculty of Biotechnology and Biomolecular Sciences, Universiti Putra Malaysia, Serdang 43400, Selangor, Malaysia

**Keywords:** bioreactor, broiler chickens, efficacy, fowl adenovirus serotype 8b, humoral and cell-mediated immunity

## Abstract

**Background and Aim:**

Fowl adenovirus (FAdV) 8b causes inclusion body hepatitis, resulting in major economic losses globally among chickens. The objectives were to inactivate FAdV 8b isolate propagated in chicken embryo liver (CEL) cells using a stirred tank bioreactor (UPM08136P5B1) and determine the humoral and cell-mediated immune response, efficacy, and virus shedding in broiler chickens.

**Materials and Methods:**

The FAdV 8b isolate UPM08136P5B1 was inactivated using binary ethyleneimine, adjuvanted with Montanide 71VG, inoculated into day-old broiler chickens in a booster group (BG) and non-booster group (NBG), and challenged with a pathogenic FAdV 8b strain. Clinical signs, gross lesions, body weight (BW), liver: body weight ratio, FAdV antibody titer using enzyme-linked immunosorbent assay, and histopathological changes were recorded. The CD3+, CD4+, and CD8+ T-lymphocyte profiles of the liver, spleen, and thymus using flow cytometry, and viral load in liver and cloacal shedding using quantitative polymerase chain reaction were evaluated.

**Results:**

Chickens in the challenged control group (CCG) exhibited mild clinical signs, gross lesions, and histopathological changes, which were absent in the inoculated groups, and had lower BW and higher liver BW ratio than chickens in the unchallenged control group (UCG); BG and NBG on 35- and 42-days post-inoculation (DPI). Chickens in NBG and BG had higher antibodies than UCG on 7, 21, 35, and 42 DPI. The challenged BG and NBG produced higher antibodies than the CCG on 35 DPI. T-lymphocytes were higher among the inoculated groups than UCG in the liver, spleen, and thymus. Inoculated challenged groups recorded higher CD3+, CD4+, and CD8+ T-lymphocytes on 35 and 42 DPI than CCG. The challenged control group had a significantly higher viral load in the liver than challenged that in BG on 35 DPI and BG and NBG on 42 DPI. The challenged control group had significantly higher challenge FAdV shedding than challenged inoculated groups on 35 and NBG on 42 DPI.

**Conclusion:**

UPM08136P5B1 was successfully inactivated and mixed with Montanide 71VG. The inactivated vaccine candidate that induced humoral and cellular immunity was effective, reduced FAdV load in the liver, and shedding in the cloaca, and could be useful against FAdV 8b infections in chickens.

## Introduction

Fowl adenovirus (FAdV) serotype 8b, which causes inclusion body hepatitis (IBH), has been reported in several countries with graded levels of economic losses [1, 2]. The control and prevention of these infections have been hampered by vertical transmission and, more importantly, due to the poor availability of specific vaccines. Most early research interest in adenovirus was mainly as a vector for other vaccines [3, 4], but its emergence as a pathogen globally has necessitated research interest in its prevention and control [5]. Due to the difficulty in treating viral diseases, vaccination has relied on their prevention and control since 1798, when Edward Jenner used cowpox to prevent human smallpox [6]. The most common protocol for the inactivated virus vaccine production involves growing these viruses in susceptible cells, embryos, or other suitable hosts and inactivating the virus mainly by chemical method – formaldehyde [7] and binary ethyleneimine (BEI) [8]. One hindrance to FAdV research *in vitro* is its difficulty in growing well in established cell lines. Efforts to grow FAdV on adult hamster kidneys, green monkey kidney cells [9], and Vero cells [5] have limitations, and chicken embryo fibroblasts are FAdV insensitive [10]. Chicken hepatoma [4, 11] and embryo kidney cells [12] showed encouraging results but with the liver being the primary predilection site for FAdV infection *in vivo* [13], CEL cells could show better adaptability for long passages. Inactivated FAdV vaccines induce a strong antibody response, although the high level of antibody response may vary depending on the age of chicks and the administration route [14]. The administration route may be related to why the neutralizing antibody response is not observed when some strains cannot cause a systemic infection following oral inoculation [15]. However, neutralizing antibodies may not be required for clinical protection against FAdVs, as shown by independent challenge protection studies [15, 16]. This has strengthened the role of cell-mediated immunity (CMI) in the limitation of FAdV infection, as supported by investigations of cytokine expression patterns, indicating a polarization toward the Th1 pathway on infection with non-pathogenic FAdV-4 and -8b strains [11]. However, the role of CMI in FAdV 8b infection in chickens is unclear. A bivalent inactivated FAdV 8b and FAdV 11 protected breeder stock, and progeny from broiler breeders vaccinated with either of the bivalents inactivated FAdV vaccines had 98%–100% survival [17]. The vaccination of broiler chickens on 17^th^ day age with inactivated FAdV 8b protected the chickens against challenges with pathogenic FAdV 8b until 70^th^ day [7].

The unavailability of commercial vaccines in the study area and elsewhere has necessitated its development to help poultry farmers deal with the burden of this disease. Again, since humoral immunity is insufficient to prevent this disease and the importance of horizontal transmission in pathogenesis, developing a vaccine that can induce cellular immunity and reduce virus shedding is ideal.

This study was conducted to inactivate the bioreactor propagated FAdV 8b isolate (UPM08136P5B1), inoculate it into broiler chickens, and determine its efficacy, humoral and CMI response, its effects on the viral load in the liver, and shedding from the cloaca.

## Materials and Methods

### Ethical approval

The animal experiment was carried out following the guidelines and ethics of the UPM Institutional Animal Care and Use Committee (IACUC) and was approved with ref number; UPM/IACUC/AUP-R086/2018.

### Study period and location

This study was conducted from March 2017 to January 2021 at the Virology Laboratory, Faculty of Veterinary Medicine, Universiti Putra Malaysia.

### Virus

Fowl adenovirus 8b (UPM08136) was isolated from an IBH outbreak in 2008 in Malaysia [18] and was used in this study. The isolate was maintained in the Virology Laboratory of the Faculty of Veterinary Medicine, Universiti Putra Malaysia, and filtered through a 0.45 mm syringe filter (Corning, USA) before use.

The FAdV 8b (UPM08136P5B1) was obtained by propagating the virus first in specific pathogen-free (SPF) chicken embryonated eggs (CEEs) through the chorioallantoic membrane one time followed by five times propagation in chicken embryo liver (CEL) cells using flasks (CELP1–P5) and one time in stirred tank bioreactor using Cytodex 1 microcarriers (P5B1). The virus isolate was identified using polymerase chain reaction (PCR), sequenced, and analyzed, as reported by Ugwu *et al*. [19].

### Challenge virus preparation

The UPM11142CELP5 isolate was passaged 2× in SPF CEE to obtain UPM11142P5EP2. The liver from dead embryos of passage 2× was macerated in PBS and centrifuged at 352× *g* for 5 min. The supernatant was filtered using a 0.45 mm syringe filter, and EID_50_ was determined. The filtrate served as a challenge virus for an experimental animal trial with a titer of 10^8^ EID_50_/mL [20].

### Inactivation of virus working seed

Thirty milliliters of 0.1 M BEI was prepared as previously described by Sarachai *et al*. [21] were added to 1.5 L FAdV 8b (UPM08136P5B1) supernatant, mixed thoroughly, incubated at 37°C for 30 h, and shaken manually hourly. After inactivation, 3 mL 1 M sodium thiosulfate, prepared as previously described by Choudhary [22], (10% of 30 mL BEI used) was added and incubated for 1 h at room temperature (30°C) to neutralize all remaining BEI [23]. Then, inactivated virus inoculum was then filtered using a 0.45 mm sterile bottle filter to remove any possible contamination and cellular debris and stored at 4°C until use. The inactivated FAdV was tested for sterility by inoculation onto confluent monolayer CEL cells, incubated at 37°C, 5% CO_2,_ and observed daily for a cytopathic effect on 7 days post-inoculation (DPI). The supernatant was used to infect the next monolayer and repeated thrice. The DNA was extracted from each passage for PCR amplification of the hexon gene as described.

### Preparation of inoculum

An aliquot of water in an oil adjuvant, Montanide ISA 71VG (Montanide), was poured into a sterile bottle and sterilized by filtering through a 0.45 mm syringe filter and stored at 30°C. The filtered Montanide adjuvant was mixed thoroughly with inactivated FAdV in a sterile bottle 1:1 (v/v) at 30°C and stored at 4°C until use.

### Immunogenicity, safety, and efficacy of inactivated FAdV serotype 8b isolate UPM08136P5B1 in commercial broiler chickens

Ninety-two, 1-day-old commercial broiler chicks were randomly divided into two main groups: The inoculated group (A) and control group (B), containing 56 and 36 chicks, respectively (Table-1). The groups were further divided into inoculated non-booster group (NBG) A1, booster group (BG) A3 and unchallenged control group (UCG) B1 subgroups containing 24, 16 and 28 chicks, respectively; and challenged non-booster, challenged booster and uninoculated challenged control groups (CCG) B2 containing 8 chicks each. All chicks were given feed and water *ad libitum*, and clinical signs were monitored daily and recorded for 42 days. On day 1 of age, corresponding to day 0 of the experimental trial, all chicks in Group A were subcutaneously inoculated with 0.5 mL inactivated FAdV isolate of UPM08136P5B1 (10^7.5^ TCID_50_/mL), whereas chicks in Group B were uninoculated. On 14-day pi, chicks in Groups A3 and A4 were subcutaneously inoculated with 0.5 mL inactivated FAdV as a booster dose. On day 28 pi, chickens in Groups A2, A4, and B2 (eight chickens each) were intramuscularly challenged with 0.5 mL UPM11142CEL5EP2 (10^8^ TCID_50_/mL).

**Table-1:** Experimental design for immunogenicity and efficacy of BEI inactivated FAdV serotype 8b isolates UPM08136CELP5B1 on commercial broiler chickens.

Groups	Time (DPI)							

	0+	7	14*	21	28#	35	42	Total
A1	-	4	4	4	4	4	4	24
A2						4	4	8
A3				4	4	4	4	16
A4						4	4	8
B1	4	4	4	4	4	4	4	28
B2						4	4	8
Total								92

BEI=Binary ethyleneimine, FAdV=Fowl adenovirus, DPI=Day post-inoculation. All chicks in the A group were inoculated with the inactivated FAdV on day-old of age (+). Booster was given to the booster group of chickens on 14 DPI (*). The chickens in the challenge groups were challenged with pathogenic FAdV on 28 DPI (#). The values in the table are number of chicks sacrificed at various intervals of time for sampling.

### Sample collection

Sampling started on day 0 pi with four chicks from the B1 group. Subsequently, four chicks were sampled from Groups A1 and B1 each on days 7, 14, 21, 28, 35, and 42 pi. Four chickens were sampled from the A3 (BG) on days 21, 28, 35, and 42 pi; and from the A2, A4, and B2 (challenged chickens) groups on days 35 and 42 pi. Clinical signs were monitored daily while BW, liver weight, and gross lesions were recorded for each chicken sampled on sampling days. Liver to BW ratio was also calculated. Each sampled chicken’s liver, thymus, and spleen samples were collected for histopathological changes and for CD3+, CD4+, and CD8+ cell phenotyping. Liver samples from all chickens were collected for the quantitative PCR (qPCR) detection of FAdV. Blood samples were collected, and serum was extracted from each chicken sampled for the measurement of FAdV antibody titer. Cloacal swabs were collected to evaluate viral shedding. The swabs were suspended in 2 mL Dulbecco’s Modified Eagle Medium (without supplements) and stored at −20°C for further processing.

### Gross lesions and histological changes

The liver, spleen, and thymus samples from each chicken in this experiment were examined for gross lesions and then fixed in 10% buffered formalin for 48 h [24]. The samples were then processed onto glass slides, stained using hematoxylin and eosin, and the slides were examined under a simple light microscope (Leica DM LB2, Germany) for histopathological changes as previously described [1]. The images were captured (Leica DFC295) and recorded.

### Enzyme-linked immunosorbent assay (ELISA) analysis of serum samples

Serum was harvested from each blood sample collected within 24 h after sample collection, centrifuged at 240× *g* for 5 min, and the serum samples were stored at −20°C until use. This test was conducted at the Laboratory of Vaccines and Therapeutics, Institute of Bioscience, UPM. The ELISA kit (BioChek, UK) was used according to the manufacturer’s recommendation and was read at 405 nm using an ELISA reader (Dynatech MR7000, USA) [25].

### Preparation of samples, staining, and flow cytometry

Three tissue samples of the liver, spleen, and thymus from each chicken group were used for immunophenotyping. A small piece of each tissue was softly macerated and filtered using a 70 μm cell strainer (FALCON-Corning, NC, USA) in a centrifuge tube and centrifuged at 376 × g for 5 min. The supernatant was discarded, and the cells were suspended in 1 mL PBS and mixed by gentle rocking, counted, and cells equivalent to 1 × 10^6^/mL from each sample were transferred to falcon tubes (FALCON-Corning) for staining. Mouse Anti-Chicken CD3-FITC [26], Mouse Anti-Chicken CD4-APC [27], and Mouse Anti-Chicken CD8α-PE [26] antibodies (SouthernBiotech, Birmingham, AL, USA) reconstituted according to the manufacturer’s recommendations were used for staining the prepared cells. Forty microliters of each antibody adequate for staining 1 × 10^6^ cells were transferred into each test falcon tube containing prepared cells and incubated at 4°C for 30 min. Then, the cells were washed with PBS (pH 7.4, 0.01 M, 4°C) thrice by centrifuging at 376 × g for 5 min at 4°C each. After washing, the cells were suspended in 500 μL PBS, sorted, assessed, and CD3+, CD4+, and CD8+ phenotypes were determined using flow cytometry on 10,000 live cells using a BD FACSCalibur flow cytometer (BD Biotec., San Diego, CA, USA) [28]. The data generated were analyzed using the CellQuest software (BD Biotec.).

### Viral genome copy number of FAdV challenge virus in the liver and cloaca of challenged chickens

#### Primer and probe design

Primers for FAdV qPCR used in this study were designed based on a partial sequence of the hexon gene of UPM11142CELP3EP2 challenge virus. Primers qHex-F 5’-GTTAGACACCACCGCACAGA-3’and qHex-R 5’-GTCACGGAACCCGATGT AGT-3’ and probe qHex Probe 5’-FAM/CCCTCCTTCTGAG TACGGAGAG-3’ BHQ1 (Microgen, Surrey, UK) were designed to detect only the challenge virus.

### Sample preparation and DNA extraction

Liver and cloacal swab samples from challenged chickens were recovered at −20°C, thawed, and mixed thoroughly by vortexing. Then, the samples were centrifuged at 352× *g* for 5 min, and 200 μL supernatant was used for DNA extraction [29]. Total DNA of each sample was extracted using an innuPREP viral DNA kit (Analytik Jena, Germany) following the manufacturer’s recommendations and yielded an average concentration of 52 ng/uL. The purity and concentration of extracted DNA from the liver and cloacal swabs of challenged chickens of each study group were determined using a spectrophotometer at a 260 nm wavelength and used as a template with primer and probe.

### Standard curve

The FAdV-positive control was used to generate the standard curve. An initial DNA concentration of 100 ng/μL was used and diluted 7-fold from 100 ng/μL to 0.0001 ng/μL. These dilutions were each amplified in triplicate in a total volume of 20 μL PCR reaction mix containing 10 μL SensiFAST™ Probe No-ROX Kit (Bioline, London, UK), 0.8 μL primer pair, 0.2 μL probe, 4.2 μL nuclease-free water, and 4 μL of the template. The non-template control was added in triplicate using nuclease-free water as a template. The qPCR amplification was conducted in CFX96™ real-time PCR Detection System (Bio-Rad, USA). The qPCR conditions were initial denaturation at 95°C for 2 min, 40 cycles of denaturation, and extension at 95°C for 5 s and 60°C for 20 s, respectively [30]. An amplification plot and standard curve with a 96% efficiency, R square of 0.997, slope value of 3.420, and y-intercept of 26.008 were obtained.

### Quantitative polymerase chain reaction amplification of extracted DNA of the liver and cloacal swab from challenged chicken

The qPCR reaction was conducted in triplicates in a total volume of 20 μL PCR reaction mix containing 10 μL SensiFAST™ Probe No-ROX Kit (Bioline), 0.8 μL primer pair, 0.2 μL probe, 4.2 μL nuclease-free water, and 1 μL of the template. The non-template control was added in triplicate with nuclease-free water as a template. The qPCR amplification was conducted in CFX96™ Real-Time PCR Detection System (Bio-Rad) [31]. The C_T_ of all replicates was obtained and the mean of each sample was determined.

### Data presentation and statistical analysis

This study’s results are presented in tables and figures. A two-way repeated measures analysis of variance on Statistical Package for the Social Sciences (SPSS) 25.0 for Windows (SPSS Inc., Chicago, IL, USA) was used to analyze the differences within and between groups with the Tukey HSD *post hoc* test at a significant p = 0.05 [32].

## Results

### Experimental animal trials

#### Clinical signs and gross lesions

No abnormal clinical signs were observed among chickens in the non-CCG as well as challenged and unchallenged NBG and BG throughout the trial. Depression and inappetence were observed within 2 days post-challenge (DPC) among chickens in the CCG, but they recovered after that.

### Body weight, liver weight, and liver-to-BW ratio (LBR)

**Figure-1:**
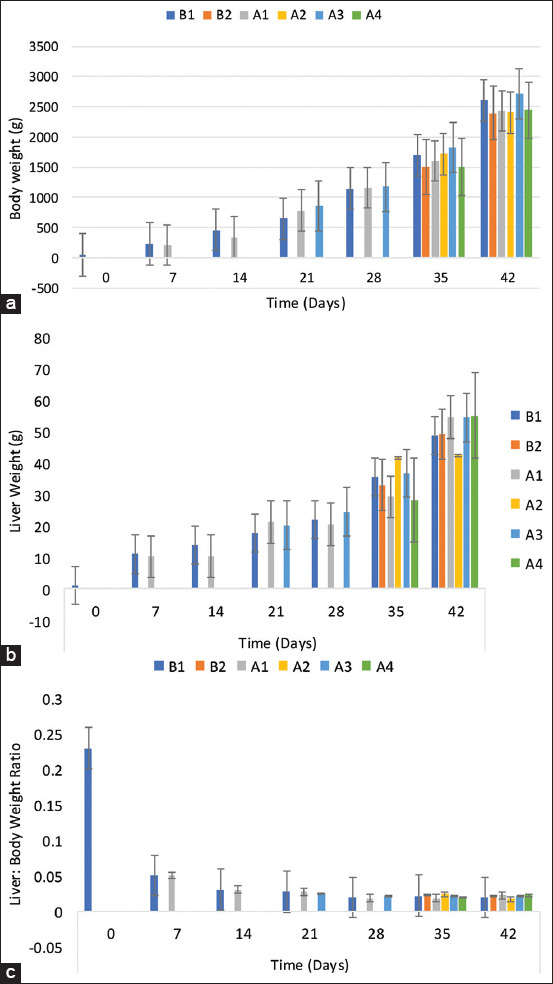
(a) Body weight, (b) liver weight, and (c) body to liver-weight ratio of commercial chickens inoculated with inactivated UPM08136P5B1 FAdV strain with Montanide 71VG adjuvant. B1=Uninoculated control, B2=Uninoculated control challenged, A1=UPM08136P5B1 without booster, A2=UPM08136P5B1 without booster challenged, A3=UPM08136P5B1 with booster, and A4=UPM08136P5B1 with booster challenged. FAdv=Fowl adenovirus.

The BW of the chickens increased to 1698 ± 72 g and 2607 ± 153 g on 35 and 42 DPI, respectively, among the control group, which was higher (p > 0.05) than that of the CCG (Figure-1a). No significant difference (p > 0.05) was observed in the chickens’ weight in NBG (A1) and the challenged NBG (A2). However, the chickens’ BW in the BG (A3) was significantly higher (p < 0.05) on 35 DPI than that of the challenged BG (A4). No significant difference (p > 0.05) was observed between the liver weight of the challenged chickens in the NBG (A2), BG (A4), and the uninoculated control group (Figure-1b). However, the chickens’ liver weight in the control group was higher (p < 0.05) than that of the chickens in the A1 group on 14 DPI. The chickens’ liver weight in the A1 was statistically similar to the control group but significantly higher (p < 0.05) than that of CCG (B2). No significant difference was observed in the LBR of chickens in the uninoculated control group (B1) and unchallenged chickens in the NBG and BG throughout the trial. However, the CCG had a significantly higher ratio than the challenged BG (Figure-1c).

### Gross lesions

No gross lesions were observed in chickens’ organs in the A1, A2, A3, A4, and B1 groups. However, the chickens in CCG (B2) showed pale, discolored, and enlarged areas in the liver in two chickens on 42 DPI and enlargement of the spleen in two chickens on 35 DPI.

### Histopathological changes

**Figure-2:**
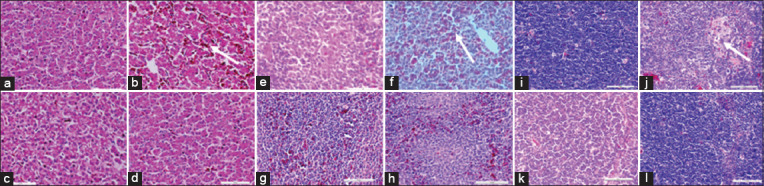
Liver, spleen, and thymus of unchallenged and challenged chickens inoculated with inactivated FAdV isolates UPM08136P5B1: (a, e, and i) Liver, spleen, and thymus of uninoculated control chickens, respectively; (b, f and j) liver, spleen, and thymus of uninoculated control challenged chickens showing necrosis (white arrow), congestion, and vacuolation of the hepatocytes; cellular vacuolation and congestion (white arrow) in spleen and lymphoid cells depletion (white arrow) in thymus, respectively; (c, g, and k) inoculated non-challenged liver, spleen, and thymus, respectively, and (d, h, and l) inoculated challenged chickens, respectively, showing normal conformity on 35 DPI. HE, 40×. FAdV=Fowl adenovirus, DPI=Day post-inoculation.

No obvious histopathological changes were observed in the liver, spleen, and thymus of chickens in the uninoculated control group (B1) and the A1, A2, A3, and A4 groups throughout the trial (Figure-2). There was mild-to-moderate necrosis, congestion, and vacuolation of the hepatocytes in the challenged uninoculated control chickens (B2 group) on day 35 DPI or 7 DPC. The spleen was congested with areas of cellular vacuolation and necrosis, while lymphoid cell depletion was recorded in the thymus (Figure-2).

### Fowl adenovirus antibody titers

**Figure-3:**
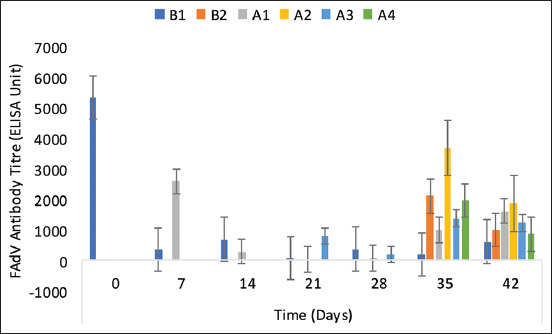
FAdV antibody titer of commercial chickens inoculated with inactivated UPM08136P5B1 FAdV strain with Montanide 71VG adjuvant. B1=Uninoculated control, B2=Uninoculated control challenged, A1=UPM08136P5B1 without booster, A2=UPM08136P5B1 without booster challenged, A3=UPM08136P5B1 with booster, and A4=UPM08136P5B1 with booster challenged. FAdv=Fowl adenovirus.

The FAdV antibody titer of the control chickens (B1 group) on day 1 of age or 0 DPI was 5353 ± 769, indicating high maternal antibody, and was 198 ± 62 and 612 ± 226 on 35 and 42 DPI, respectively (Figure-3). The inoculated NBG had higher antibodies on 7, 21, 35, and 42 DPI, indicating that inactivated UPM08136CEL5B1 with Montanide 71 VG adjuvant-induced antibodies in commercial chickens, while among the challenged chicken groups, the NBG recorded higher FAdV antibody on 35 DPI, which showed the response of the inoculated chickens on pathogenic FAdV challenge.

### CD3+ T-lymphocyte sub-population in the liver, spleen, and thymus

**Figure-4:**
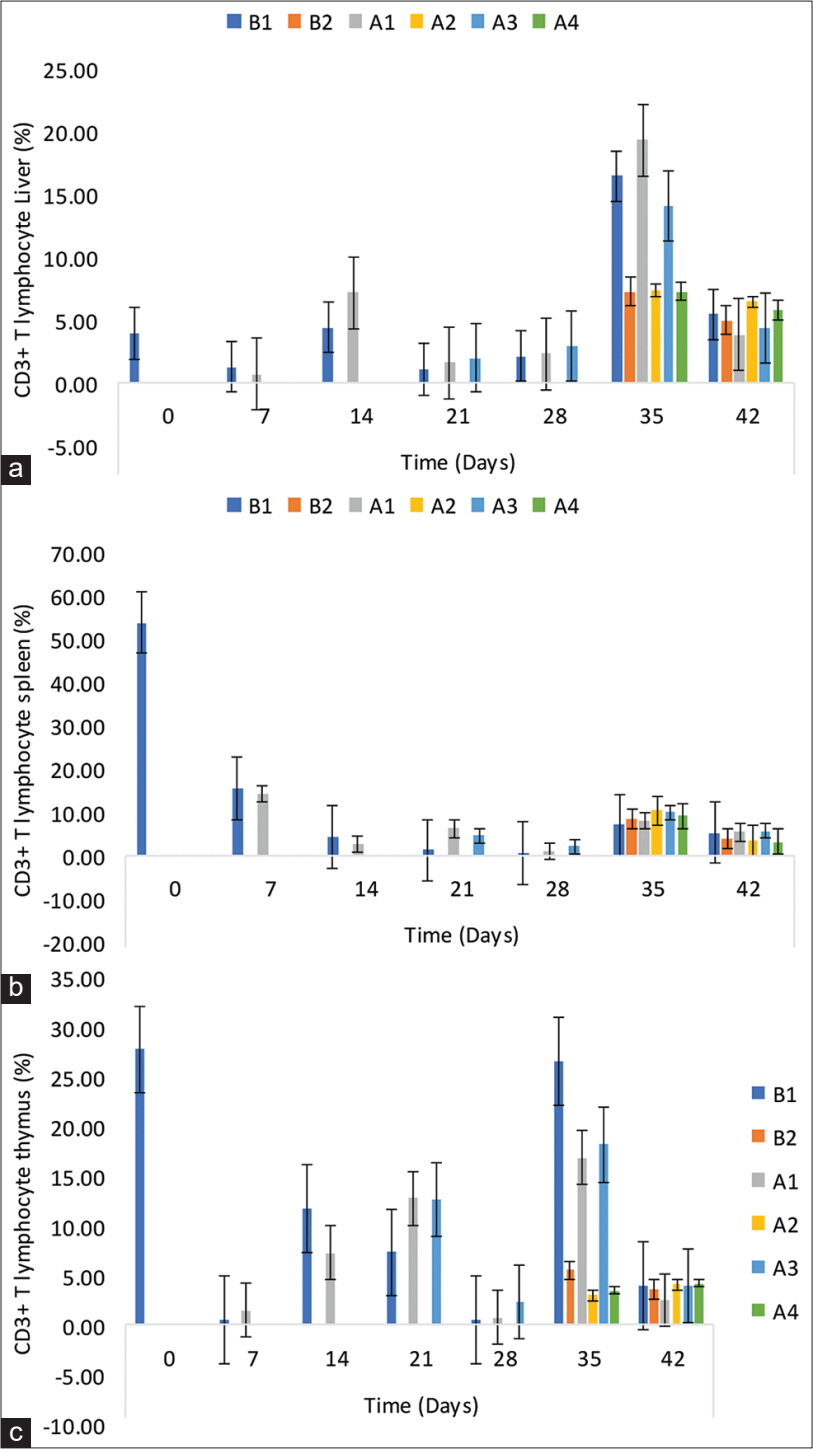
CD3+ T-lymphocyte subpopulation in the (a) liver, (b) spleen, and (c) thymus of commercial chickens inoculated with inactivated UPM08136P5B1 FAdV strain with Montanide 71VG adjuvant. B1=Uninoculated control, B2=Uninoculated control challenged, A1=UPM08136P5B1 without booster, A2=UPM08136P5B1 without booster challenged, A3=UPM08136P5B1 with booster, and A4=UPM08136P5B1 with booster challenged. FAdv=Fowl adenovirus.

The percentage of CD3+ T-lymphocytes in the liver of the uninoculated control chickens (B1 group) was 3.90 ± 1.19, 1.23 ± 0.93, 4.40 ± 0.32, 1.03 ± 0.43, and 2.10 ± 0.36 on 0, 7, 14, 21, and 28 DPI, respectively (Figure-4a). The percentage of CD3+ T-lymphocytes in the liver of challenged control chickens (B2 group) on 35 DPI was significantly lower (p < 0.05) than that of the unchallenged control B1 chickens. The percentage of CD3+ T-lymphocytes in the liver of challenged chickens (A2 group) was significantly lower (p < 0.05) than that of the A1 group chickens. In the spleen, there were higher percentages among the NBG and BG than the control group on 21, 35, and 42 DPI (Figure-4b). In the thymus, there was a significant reduction in the CD3+ sub-population among the challenged chickens of all groups on 35 DPI (Figure-4c).

### CD4+ T-lymphocyte sub-population in the liver, spleen, and thymus

**Figure-5:**
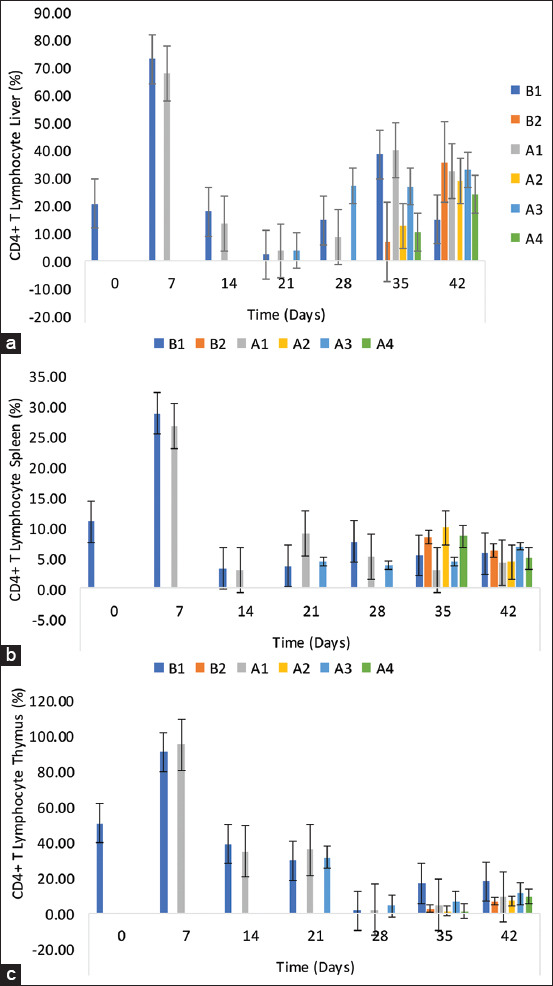
CD4+ T-lymphocyte subpopulation in the (a) liver, (b) spleen, and (c) thymus of commercial chickens inoculated with inactivated UPM08136P5B1 FAdV strain with Montanide 71VG adjuvant. B1=Uninoculated control, B2=Uninoculated control challenged, A1=UPM08136P5B1 without booster, A2=UPM08136P5B1 without booster challenged, A3=UPM08136P5B1 with booster, and A4=UPM08136P5B1 with booster challenged. FAdv=Fowl adenovirus.

The percentage of CD4+ T-lymphocytes in the chickens’ liver was 20.9 ± 1.19, 73.07 ± 6.68, 17.70 ± 3.25, 2.90 ± 0.26, 14.60 ± 3.52, 38.47 ± 10.14, and 14.70 ± 1.98 on 0, 7, 14, 21, 28, 35, and 42 DPI, respectively, among unchallenged control chickens (B1 group) (Figure-5a). The inoculated NBG and BG recorded higher percentages on 21, 35, and 42 DPI. A significant reduction was observed in the percentages of CD4+ cells in the liver of challenged chickens on 35 and 42 DPI in all groups compared with their unchallenged groups. The challenged control chicken group had a lower percentage than the challenged NBG and BG. The percentage of CD4+ T-lymphocytes in the spleen was higher among the inoculated challenged groups than in the CCG. This was also higher among the challenged NBG and BG than their unchallenged counterparts (Figure-5b). In the thymus, the CD4+ T-lymphocytes were statistically similar throughout the trial among all groups except on 35 DPI where the inoculated groups (NBG and BG) were lower (p < 0.05) and on 42 DPI where the challenged groups (NBG and BG) were higher (p > 0.05) than the CCG (Figure-5c).

### CD8+ T-lymphocyte sub-population in the liver, spleen, and thymus

**Figure-6:**
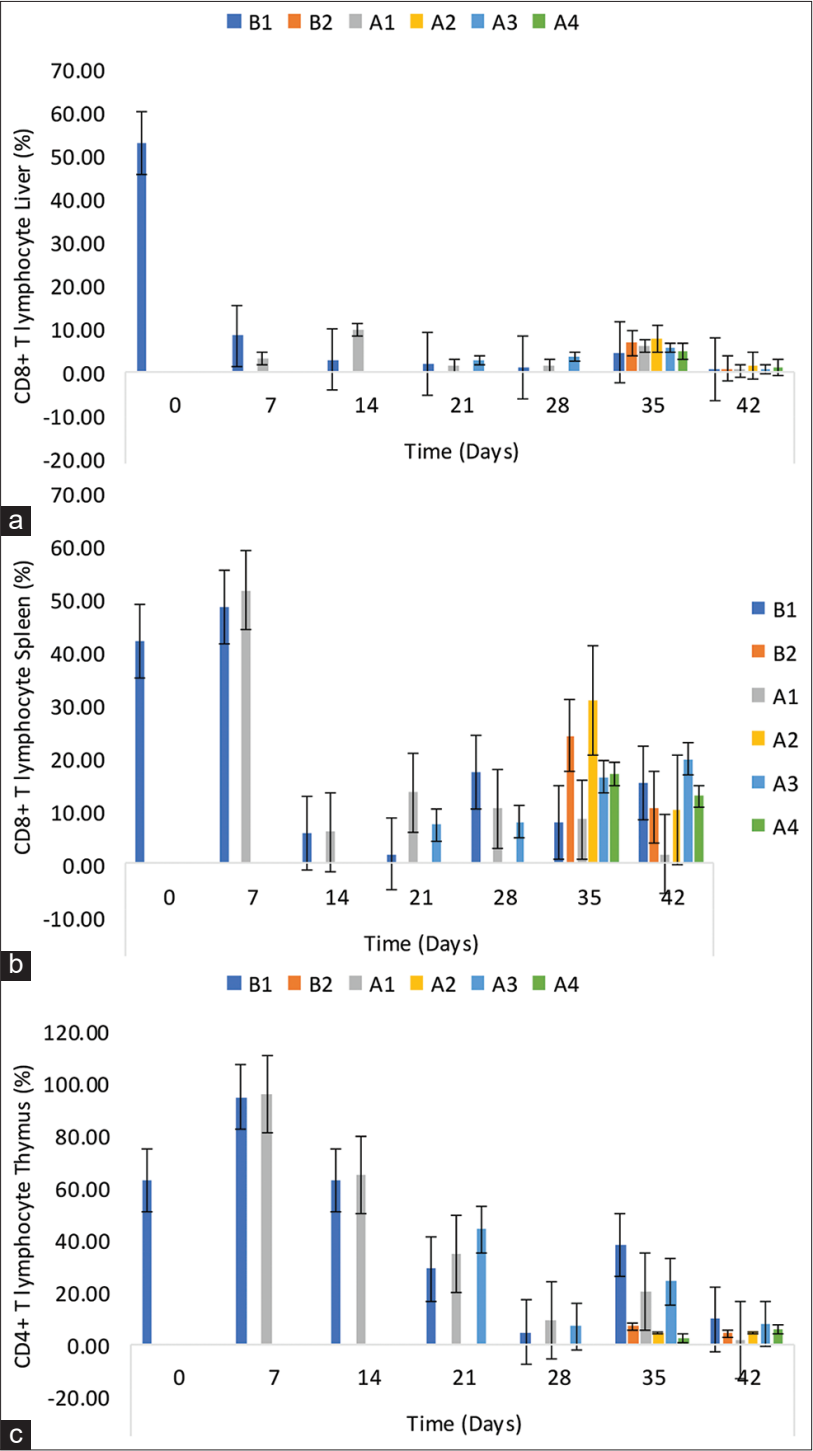
CD8+ T-lymphocyte subpopulation in the (a) liver, (b) spleen, and (c) thymus of commercial chickens inoculated with inactivated UPM08136P5B1 FAdV strain with Montanide 71VG adjuvant. B1=Uninoculated control, B2=Uninoculated control challenged, A1=UPM08136P5B1 without booster, A2=UPM08136P5B1 without booster challenged, A3=UPM08136P5B1 with booster, and A4=UPM08136P5B1 with booster challenged. FAdv=Fowl adenovirus.

The percentage of CD8+ T-lymphocytes in the liver of inoculated chickens was significantly higher (p < 0.05) on 14, 21, 21, 28, and 35 DPI than the uninoculated control chickens (B1 group) (Figure-6a). No statistical difference was observed in the percentages between the challenged and unchallenged across groups. The percentage in the spleen was higher (p < 0.05) on 21, 7, 7, 14, 35, and 42 DPI (Figure-6b). The challenged NBG and BG values were higher on 35 and 42 DPI, respectively than the CCG. The challenged chickens had a higher percentage on 35 DPI than the non-challenged groups. In the thymus, there was a higher CD8+ cell population in the NBG and BG chickens on 7, 14, 21, and 28 DPI (Figure-6c). There was a significantly higher percentage among unchallenged chickens than the challenged chickens.

### Viral genome copy number of FAdV challenge virus in the liver and cloaca

**Figure-7:**
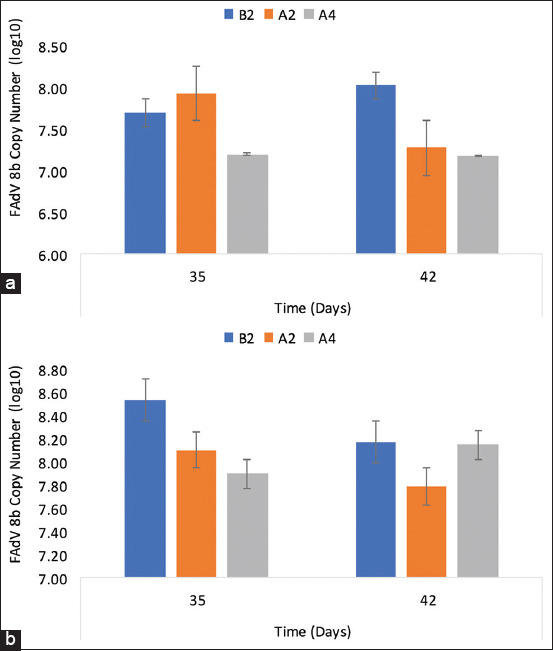
Viral genome copy number of FAdV challenge virus in the (a) liver and (b) cloaca of challenged chickens. B2=Uninoculated control, A2=UPM08136P5B1 without booster, and A4=UPM08136P5B1 with booster groups. FAdv=Fowl adenovirus.

**Figure-8:**
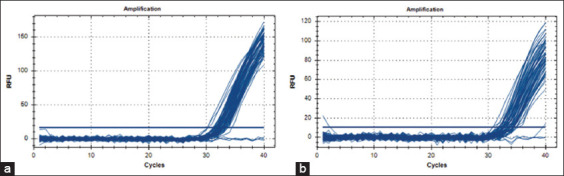
Amplification plot of the probe-based qPCR of viral genome copy number in the (a) liver and (b) cloaca of chickens infected with FAdV 8b challenge virus. FAdV=Fowl adenovirus.

The copy number of challenge FAdV in the liver of challenged chickens in the B2 group was log (7.71 ± 0.07) on 35 DPI and log (8.03 ± 0.11) on 42 DPI, significantly higher (p < 0.05) than that of the inoculated chickens in the A2 and A4 groups (Figure-7a), as shown in the amplification plot (Figure-8a), which showed reduced proliferation of the challenge virus in the liver of inoculated chickens.

On 35 DPI, 7 DPC, the copy number of challenge FAdV in the cloaca of challenged uninoculated control chickens (B2 group) was log (8.54 ± 0.01), which was significantly higher (p < 0.05) than the inoculated and BGs, and was log (8.17 ± 0.04) on 42 DPI (14 DPC). This was significantly higher (p < 0.05) than A2 and higher (p > 0.05) than the A4 groups, indicating a reduced virus shedding among the inoculated (Figure-7b), as shown in the amplification plot (Figure-8b).

## Discussion

Fowl adenovirus serotype 8b isolate (UPM08136P5B1) was successfully inactivated with BEI and was adjuvanted with Montanide 71VG. An advantage of BEI is that it does not interfere with the antigenicity of the viral antigens [33]. Conversely, Montanide 71VG is comparatively stable, easy to inject, with minimal side effects, and induces strong and long-term immunity [34] even with highly reduced antigens [35].

The inactivated FAdV serotype 8b with Montanide 71VG induced humoral and interestingly CMI, which protected the chickens against challenge with pathogenic FAdV serotype 8b in both the BG and NBG. This is remarkable because inactivated vaccines are unassociated with cellular immunity [17, 36]. No mortality was observed in the trial among the inoculated, the uninoculated, and the challenged chickens. Low or no mortality in broilers intramuscularly inoculated with FAdV-8b at 2 weeks of age has been reported [37]. However, chickens infected on 1–4 days of age were reported to have high mortality [38], supporting the impact of age, genetic background of the bird, experimental versus field condition, inoculation route on FAdV pathogenicity, and IBH manifestation [37]. Experimental infection of chickens with FAdVs rarely causes clinical, gross, and histopathological changes similar to that experienced with the same virus under natural circumstances [39]. Following the report of Adair and Fitzgerald [40] that FAdVs rarely cause disease in adult chickens, it is only probable that age, among other underlying factors, including maternally derived antibodies, may have played a role in limiting the exhibition of severe clinical signs and mortality. However, the presence of mild clinical signs in the uninoculated challenged group, which were absent among the inoculated challenged groups, could indicate the protective efficacy of the inactivated FAdV 8b strains with Montanide 71VG adjuvant.

The safety of the inactivated vaccines and their boosters was shown by the lack of clinical signs and lesions in the inoculated chickens, similar to the uninoculated control group. However, there was a pale, discolored, and enlarged liver, spleen, and thymus involving a few chickens in the challenged uninoculated control group, which were not found in the inoculated challenged chickens (with and without BGs). These findings were similar to the report of Chen *et al*. [41] in whose experimental trial showed no clinical signs or necrotic lesions among chickens inoculated with 2 × 10^6^ TCID_50_ pathogenic FAdV 8a through the intramuscular route. However, they reported hemorrhaging in the liver. Cui *et al*. [42] reported liver enlargement and splenomegaly in chickens inoculated with FAdV 8a. The presence of some lesions in the liver, spleen, and thymus among chickens in the uninoculated challenged group and the absence of such lesions in the inoculated challenged group is evidence of protective immunity acquired by chickens in the inoculated group. The lack of histopathological changes in the liver, spleen, and thymus of inoculated chickens and the uninoculated control chickens indicates that the BEI inactivated FAdV 8b delivered with Montanide 71VG was well tolerated in the system of commercial broiler chickens. However, the presence of changes in the organs of the uninoculated control chickens, which were mostly spontaneous and ungeneralized, is a sign that it is safe and shows that the inactivated FAdV serotype 8b in this trial protected the chickens against infection and tissue damage associated with FAdV 8b infections.

The BW of the uninoculated control group was higher than that of the inoculated groups on 7 and 14 DPI, similar to the previous reports [43]. No significant difference was observed between the weights of the uninoculated control group and that of the inoculated groups, indicating that the inactivated FAdV was safe and well tolerated. The lower body weight recorded by the uninoculated control challenged chickens in the B1 group than that of the unchallenged control chickens and the inoculated challenged chicken groups showed that the challenge virus limited the normal growth rate of the uninoculated chickens, highlighting poor growth associated with FAdV infections [40]. The normal and lower LBRs observed among uninoculated control chickens and inoculated non-challenged chickens show that the BEI inactivated FAdV with Montanide 71VG adjuvant is safe and well tolerated among commercial broiler chickens. Since liver enlargement is one of the most striking gross lesions associated with FAdV infections in chickens [44]. The higher ratio among the uninoculated CCG shows that the inoculated chickens may have been protected from inflammatory reactions in the liver. This means that the inoculation with inactivated FAdV 8b with Montanide 71VG helped reduce the effect of the pathogenic FAdV 8b challenge on the inoculated groups.

The antibody titers recorded in this trial on day 1 of age or day 0 post-inoculation showed that the experimental chickens had high maternally derived antibodies (MatAb). However, due to the short half-life of MatAb, which may last only 3–5 days, their protection lasts up to 2 weeks and then decreases below protective levels [45]. This conforms to the result of this trial in which the antibody titer of the uninoculated control chickens decreased significantly on 7 and 21 DPI, respectively. While MatAb protects progeny in early life, they suppress a full response to vaccination at a young age [45]. Such suppression of antibodies and cellular immune responses has been reported for various viruses [46, 47]. Fowl adenovirus induces passive immunity, which interferes with vaccination [22, 48]. The interference by MatAb in the presentation of antigens to B cells, which should trigger antibody production, is very limiting to inactivated vaccines, particularly because it is presented extracellularly [49]. In this trial, high antibodies were induced among the inoculated groups, which were much higher than that in the uninoculated control on 7, 35, and 42 DPI. However, the titer of the inoculated may have been affected by the high MatAb. However, on the challenge, there was a highly increased antibody titer in all groups. While antibodies decreased for the inoculated groups on 42 DPI, the viral load in the liver also decreased on 42 DPI, which could establish a relationship between antibody titer and viral load in this trial. It seems that the antibody titer became lower on 42 DPI among the groups that had lower virus load and replication and higher in those that still had replication and high load, as observed with uninoculated control challenged chickens. Interestingly, the high antibody titer among the challenged uninoculated control group did not translate to low viral load in the liver, indicating that their antibody titer may be unspecific. In contrast, the antibody titer of inoculated groups was associated with a significantly lower viral load. This was more prominent among the A3 (booster) group, meaning that the chickens inoculated with BEI inactivated UPM08136CELP5B1 were protected from the pathogenic effects of the FAdV challenge in the liver. By limiting the replication of the challenge virus in the liver of the inoculated, the inactivated FAdV serotype 8b adjuvanted with Montanide 71VG has proven effective and could be used as a vaccine against the FAdV 8b challenge.

For a vaccine to be considered effective against a viral disease, it should also be capable of inducing sufficient amounts of CMI, particularly CD8+ cells, to kill virus-infected cells [50]. This corroborates with the report of a good protective immune response to challenges associated with FAdV serotype 8b vaccines, even with low neutralizing antibody titer [15]. It is only probable that other factors, including CMI, may have contributed to the protective efficacy of the FAdV vaccines on chickens against challenge with pathogenic strains. To achieve this, inactivated vaccines traditionally unassociated with CMI inducement [50] are usually aided by appropriate adjuvants [51]. In this trial, BEI inactivated FAdV 8b, with Montanide 71VG adjuvant, induced cell-mediated immune response. The undulation in the CD3+ T-cell sub-population in the inoculated chickens is a sign of T-lymphocyte inducement, an essential requirement for overcoming viral infections [51]. Montanide 71VG induced high cellular immunity when coupled with inactivated avian influenza virus vaccine in chicken [52] and could have aided the inducement of CMI in this trial. The CD3+ protein complex was first identified by immunoprecipitation experiments using human T-cells [53] and is a defining feature of the T-cell lineage; therefore, anti-CD3 antibodies can be used effectively as T-cell markers [53]. There were also higher CD4+ cells in the liver among the chickens, which indicates that the inactivated FAdV 8b with Montanide 71VG excited high immunological competency in the inoculated chickens, thereby preparing the chickens for the FAdV challenge. The CD4+ cells, which play a modulatory role in CMI, also function to ensure optimal responses by other lymphocytes [54]. The CD4+ T-cells are necessary as helpers to promote B-cell antibody production and are often required for generating cytotoxic and memory CD8^+^ T-cell populations in addition to other roles for CD4+ T-cells in enhancing innate immune responses and in mediating non-helper antiviral effector functions [54]. While the CD8^+^ cell sub-population decreased on 14 DPI among the uninoculated control chickens, it increased among the inoculated chickens, which could be due to the declining effects of maternally derived immunity, indicating that the cells responsible for virus clearance in the liver of chickens infected with FAdV 8b by killing virus-infected cells [55] were induced pre-challenge. Upon infection, viruses will synthesize their own proteins through the protein synthesis mechanisms of the host cell. Some of these newly synthesized proteins will degrade into peptide fragments, which, on having enough binding affinity, will bind to MHC Class I molecules and form MHC Class I-peptide complexes that will be presented on the cell surface of an infected cell. The CD8^+^ T-cells activated through the costimulation efforts of dendritic and CD4+ T-cells are specific for the peptide, and can recognize the MHC Class I-peptide complex, thereby inducing apoptosis of the infected cell by releasing cytotoxic granules [56]. This specificity could explain why despite the expression of CD8+ T-cells, uninoculated control chickens still had significantly higher morbidity of the disease and higher viral load in the liver than chickens in the inoculated groups. In the spleen, CD3+, CD4+, and CD8+ T-lymphocytes were induced in the inoculated chicken groups. During embryonic development, the immature B- and T-lymphocytes are present in the bursa and thymus, respectively [57]. Although T-cells differentiate in the thymus, they migrate during the 3^rd^ week of embryo formation from the thymus to the peripheral lymphoid system, including the spleen, and lymphoid cell aggregates start to colonize the gut, the trachea, and the esophagus and can be found at the Harderian gland and the cecal tonsils at hatch [58]. Lymphocytes also develop in the white pulp of the spleen, which is the organ of the interface between peripheral blood and pathogens, and is very important in the immune response to viral infections. With the upregulation of lymphocytes in the spleen among the inoculated groups, especially when challenged, memory T-cells have probably been developed, which is very important for the protective efficacy of vaccines [59]. In the thymus, the CD8+ cells were similar in population to the CD4+ cells, although the population of CD8+ cells was slightly higher. Among the inoculated, the percentage of CD8+ cells and the viral load in the liver following the challenge seems to be related. Inoculated group A3, with the lowest percentage, had the lowest copy number on 35 DPI, showing a relationship between CD8+ cells sub-population and viral load in the liver following the challenge. This might be due to the cytotoxic activity of the CD8+ cells. In contrast, despite high CD8+ cells among the uninoculated control, they still had the highest copy number of FAdV challenge virus in the liver, suggesting unspecificity. The decrease experienced among the challenged compared with the unchallenged could also be attributed to lymphoid depletion, but this was more prominent among the uninoculated. With the histopathological changes observed in the thymus among the uninoculated challenged chickens, the inoculated group was certainly protected from lymphoid depletion.

The chickens in this trial shed the FAdV challenge virus for up to 2 weeks post-challenge, which was similar to other FAdV infection reports [60]. After infecting day-old SPF chickens with FAdV through the oral route, the virus was still detected in the gut at 12 weeks post-infection [61]. However, the quantity and duration of virus shedding in the feces are more related to the dose of the virus inoculated than to the pathogenicity of the virus [36, 42]. The significantly higher (p < 0.05) shedding of FAdV challenge virus among the uninoculated challenged groups than all the inoculated challenged groups means that the inactivated FAdV inoculations administered with and without booster provided blocking immunity to the chickens. The indication of potential vaccine efficacy can be shown by the ability of the vaccine to reduce or stop the shedding of the virus in the environment [62], meaning that the vaccine protected chickens and prevented future infections. Vaccine’s ability to protect against clinical disease is important, but despite billions of doses of Newcastle disease virus vaccines administered to poultry worldwide, the disease is still endemic in many countries, indicating that it is necessary to redirect the focus to include the reduction of shedding, which has become pertinent in preventing and eradicating viral diseases [63]. The frequency and duration of production of vaccine immunity should be measured by the vaccine’s ability to reduce viral shedding [64]. In this study, a booster administration had a statistically similar effect on viral shedding with non-booster, which agrees with a previous report where repeated vaccination, although reduced shedding in the H3N2 influenza virus, did not produce a better result than single vaccination [65].

## Conclusion

Pathogenic FAdV 8b (UPM08136) isolate propagated in stirred tank bioreactor was inactivated, adjuvanted with Montanide 71VG, and inoculated into commercial broiler chickens to determine its efficacy, immunogenicity, and virus shedding. The BEI inactivated UPM08136CELP5B1 administered singly on day old or with a booster on 14 DPI induced both humoral and CMI, which protected commercial broiler chickens from pathogenic FAdV challenge, reduced viral load in the liver and the viral shedding. Thus, UPM08136CELP5B1 isolate has been demonstrated to possess strong potential as a vaccine candidate and is recommended to be used in broiler chickens against FAdV 8b infections.

## Authors’ Contributions

MH, ARO, and AI: Conceptualization. CCU, MIN, and ARO: Formal analysis. CCU: Methodology. MH, ARO, MIN, and AI: Supervision. CCU: Writing – original draft. CCU, MH, ARO, MIN, and AI: Writing – review and editing. All authors have read and approved the final manuscript.
